# The impact of economic agglomeration on China’s urban public health

**DOI:** 10.3389/fpubh.2024.1476339

**Published:** 2024-09-09

**Authors:** Honghua Wu, Chen Li

**Affiliations:** ^1^School of Economics, Wuhan Donghu University, Wuhan, China; ^2^School of Management, Shanghai University of Engineering Science, Shanghai, China

**Keywords:** urban public health, economic agglomeration, bidirectional fixed effects model, pilot of tight city medical group, China

## Abstract

**Introduction:**

This study aims to explore the impact of economic agglomeration on the urban prosperity through economies of scale and agglomeration, it may also affect the public health of the agglomeration area.

**Methods:**

This paper takes 280 cities in China as the research object, and explores the impact of economic agglomeration on public health through a two-way fixed effects model, instrumental variable method, and generalized moment estimation.

**Results:**

The results indicate that: (1) the improvement of China’s economic agglomeration can significantly promote urban public health, and economic agglomeration is a prerequisite for the improvement of urban public health, but there is no reverse causal relationship. (2) The enhancement of economic agglomeration in Northeast China has the greatest promotion effect on public health, followed by the eastern, western, and central regions; The economic agglomeration enhancement of the pilot medical group in closely connected cities has a greater promoting effect on public health than the pilot medical group in non-closely connected cities. (3) Empirical results based on micro sample data show that the improvement of economic agglomeration will also promote the increase of the number of public hospitals in cities.

**Discussion:**

This study emphasizes the important role of economic accumulation in the improvement of urban public health and provides empirical support for future economic development policies and practices.

## 1 Introduction

The proportion of the global urban population has increased rapidly from 25% in 1950 to about 50% in 2020. But growth is expected to slow over the next 50 years, increasing to 58% in 2070. Since the reform and opening up, China has undergone the fastest and largest industrialization and urbanization process in the world. By the end of 1978, there were only 170 million permanent urban residents in China, and the urbanization rate of permanent urban residents was only 17.92%. By the end of 2022, the number of permanent urban residents had reached 920 million, 750 million more than that at the end of 1978, or an average annual increase of 17.04 million. The urbanization rate of permanent residents reached 65.2%, an increase of 47.28% points over the end of 1978. With the huge changes in urban environment and residents’ lifestyles, many environmental risk factors and new health diseases pose a serious threat to national health. Under the influence of the global strategy for healthy cities, in order to address complex public health risks, China has continuously proposed health intervention strategies such as the “Guiding Opinions on the Construction of Healthy Cities and Healthy Villages (2016)” and the “Healthy China 2030’’ planning outline, taking the construction of “healthy cities” as an important lever to promote the construction of “Healthy China.” At the same time, thanks to the effective spatial agglomeration of economic activities, the Chinese economy has achieved sustained and rapid growth, and the spatial agglomeration process of economic activities is further strengthening. Cities are the core carriers of economic agglomeration development and promoting the construction of a healthy China. While economic agglomeration promotes urban prosperity through economies of scale and agglomeration effects, it may also directly or indirectly affect the public health of agglomeration areas through wealth creation, technological progress, resource supply, population mobility, environmental pollution, and other means (1–3). Therefore, how to integrate the concept of health intervention into the entire process of urban and rural planning, construction, and governance through the rational allocation of public health resources, promote the coordinated development of urban and people’s health, and explore whether and how to “move toward health in agglomeration” from the urban scale is an urgent and significant issue in front of the academic community.

The essence of enhancing economic agglomeration is to fully leverage the leading, driving, and radiating role of large cities, and improve the level of public health resource agglomeration and the scope of urban spatial services (4). Therefore, economic gatherings can affect urban public health through the following three pathways. One is the agglomeration effect. With the continuous improvement of economic agglomeration, various types of public health factors are rapidly gathering in urban spaces, forming economies of scale effects. At the same time, the flow of a large number of specialized medical practitioners between cities also helps to disseminate implicit public health knowledge, promote knowledge spillover and technology transfer. Other hospitals and institutions within the agglomeration area can imitate and learn new technologies without having to invest in high research and development costs, thereby contributing to the improvement of urban public health (5–8). Therefore, the improvement of economic agglomeration will promote the level of public health agglomeration, thereby achieving externalities in knowledge production and promoting the improvement of public health. The second is cost-effectiveness. In the Internet era, there is a significant diminishing marginal cost effect in the production process of cities. With the increase of cities’ position in the economic agglomeration system and production scale, the cost of public health will also decrease, thereby promoting larger scale specialization and division of labor (9, 10). Under the framework of monopolistic competition, it will lead to an increase in total output. The third is resource flow effect. Similar to traditional economic factors, public health resources also have the characteristics of scarcity and pursuit of maximizing their own value, and will flow from cities with low marginal returns to cities with high marginal returns. The different administrative levels of cities will to some extent affect the regional allocation of urban public health resources, especially the cross regional flow of high-end medical talents. In order to pursue the maximization of their own interests, high-end medical talents will migrate between cities through “voting with their feet,” that is, they will move to cities with more development opportunities, better scientific research environment, and more generous welfare benefits (6, 11–14).

Currently, there are not many direct relevant literature on the public health effects of economic agglomeration, and similar results are mainly limited to the impact of a specific industry’s spatial agglomeration process on a certain dimension of public health, which is quite different from the research topic (15, 16). However, by reviewing the literature, one can also find indirect theoretical evidence of the impact of economic agglomeration on public health. For example, economic agglomeration can improve residents’ material living conditions, increase the supply of public health resources, and exacerbate haze pollution, which undoubtedly may further affect public health (4, 17–22). Although these studies have paid attention to the differences in public health among cities of different levels and sizes, they have not conducted in-depth research on the relationship between economic agglomeration and urban public health, nor have they revealed the mechanism of economic agglomeration on urban public health. Existing literature provides rich insights for research work, but the research is also quite inadequate. First, due to the continuous improvement of science and technology and medical and health conditions, the frequency of large-scale outbreaks of public health problems and their direct impact on macroeconomic growth is not high. Most scholars have not been able to predict that public health problems in today’s era may also slow down economic development, and therefore have not conducted in-depth mechanism analysis and systematic empirical investigation into the economic drivers of public health problems. Secondly, when conducting relevant empirical research, most literature does not fully consider the possible bidirectional causal relationship between core explanatory variables and public health, which may lead to endogenous problems. It also fails to pay enough attention to the possible spatial correlation characteristics of public health (such as inter-city transmission of diseases), and does not thoroughly reveal the heterogeneity information of research objects in terms of economic agglomeration patterns, location characteristics, and urban hierarchy. This may result in biases in estimated parameters and real parameters, and prevent scientific and precise policy guidance for different regions based on local conditions. Thirdly, existing literature measuring public health often simply focuses on a single indicator to seek a proxy variable to characterize it, ignoring the multidimensional and comprehensive characteristics that public health itself should have. Such a simplistic approach cannot better fit the realities of public health. Therefore, the innovation of this paper is as follows: first, this paper explores the economic drivers of public health problems in depth, linking economic agglomeration with public health; Second, this paper fully considers the bidirectional causal relationship between the core explanatory variables and public health, as well as the spatial correlation characteristics of public health. Third, considering the multi-dimensional and comprehensive characteristics of public health, this paper constructs a comprehensive index of urban public health, rather than a single variable representation.

Obviously, public health is closely related to urban needs. Cities are the spatial carriers of high-quality production factors. High-quality public health resources are always concentrated in large cities, and then spill over to small and medium-sized cities. In addition, urban development itself has become an important source of public health needs (21). However, existing research has rarely addressed the relationship between economic agglomeration and public health. Does economic agglomeration pose a restrictive condition on public health? If so, what mechanism does it use to affect public health? Is there a nonlinear relationship between economic agglomeration and public health at different city sizes? Is there an optimal economic agglomeration that promotes public health improvement? In response to the above problems, this study will take 280 cities in China as the research object, conduct a systematic study on the relationship between economic agglomeration and public health, empirically analyze the impact of economic agglomeration on public health through a two-way fixed-effect model, and use instrumental variable two-stage least squares estimation (IV-2SLS), generalized method of moments estimation (GMM) and other methods to weaken the impact of endogenous factors on the estimation results.

## 2 Econometric model and variable selection

### 2.1 Econometric model

Based on the analysis above, this paper focuses on revealing the mechanism and degree of the impact of economic agglomeration on public health in the empirical part, and establishes the following panel least squares regression model:


(1) $$\mathrm{Phealt}h_{\mathrm{it}}=\beta_{1}Urbh_{\mathrm{it}}+\beta X_{\mathrm{it}}+\beta_{0}+T+\alpha +\alpha_{\mathrm{it}}$$

In the formula, *Phealth_it_* represents urban public health, *Urbs_it_* represents economic agglomeration, *X_it_* is the control variable, *T* is the time fixed effect, *α* is the individual fixed effect, *ɛ_it_* is the random interference term, *β*, *β*_0_, *β*_1_ are the estimated coefficients.

### 2.2 Urban public health (*Phealth*)

Although the academic community generally believes that public mental health, social pressure, and moral quality are all important factors in public health, due to data availability constraints, existing research often seeks proxy variables from the dimension of public physical health to represent public health, and often uses a single negative measurement indicator (23–26). This approach is reasonable, as modern medicine has long established that mental health, social stress, and moral character significantly determine an individual’s health level. Therefore, public health status is the most intuitive representation of public health, but using a single negative indicator to measure public health can be biased. Therefore, the paper is consistent with most literature, focusing on the health status of urban residents in terms of physical function, while taking into account both the necessary basic support conditions and the practical improvement of health performance when measuring public health, to construct a composite index of urban public health. On the one hand, the necessary public health foundation is the logical starting point and prerequisite for improving urban public health (27–30). Three indicators, namely the number of doctors per 10,000 people, *per capita* fiscal expenditure on medical and health care, and the number of beds in medical and health institutions per 10,000 people, are selected to characterize it from the perspective of “human, financial, and material” investment. On the other hand, the fundamental value pursuit of urban public health is to increase the “expected output” related to health as much as possible and reduce or eliminate the “undesired output” as much as possible under the established public health infrastructure support conditions. The incidence rate of population survival reflects the “expected output,” while the incidence rate of infectious diseases reflects the “undesired output.”From the perspective of history and reality, large-scale outbreaks of public health problems are more often presented in the form of infectious disease epidemics. The conflict between high population density brought about by economic agglomeration and the social distance required for infectious disease prevention and control often poses a greater risk of epidemic in the agglomeration area. The ability of the agglomeration area to prevent and control infectious diseases is also a direct reflection of its own public health governance capacity. Therefore, the incidence rate of infectious diseases is more suitable for fitting the current situation of urban public health than other negative indicators. This study is based on the incidence rate data of provincial-level Class A and B legally reported infectious diseases. The provincial data is weighted by the proportion of urban population size and the proportion of urban financial expenditure on medical and health care (reverse weight), respectively, to estimate the incidence rate of infectious diseases at the city level. The fixed-base range entropy weight method is used to calculate the urban public health composite index. The advantage of this method is that it can objectively assign corresponding weight values to different indicators with different levels of contribution, and fully reflect the spatial and temporal dual-dimensional and dynamic comparable characteristics of the evaluation object. For specific calculation steps, please refer to the literature.

### 2.3 Economic agglomeration (*Urbh*)

Drawing on the research of Wang et al. (31), an indicator evaluation system for economic agglomeration is constructed from the aspects of consumption scale, resource energy consumption level, degree of opening up to the outside world, manufacturing level, employment scale, and infrastructure level. Nine indicators are selected to evaluate economic agglomeration, including total retail sales of consumer goods in cities (in 10,000 yuan), total electricity consumption in the whole society (in 10,000 kWh), *per capita* electricity consumption (in kWh/person), actual utilization of foreign capital (in 10,000 US dollars), the proportion of actual utilization of foreign capital in the gross regional domestic product (%), the number of employees in the tertiary industry (in 10,000 people), the passenger volume of civil aviation (in 10,000 passengers), the proportion of passenger volume of civil aviation in the population (%), and the proportion of passenger volume of highway and waterway in the population (%). In addition to testing the correlation, it will also investigate whether economic agglomeration has a causal relationship with public health. Therefore, it is not enough to simply rank economic agglomeration after assigning values, but also to examine the impact of changes in economic agglomeration on public health. Comprehensive use of principal component analysis and entropy method to assign weights, and use least squares to minimize the deviation between the final determined weights and the two methods.

### 2.4 Control variables

Drawing on existing literature on the form of the health production function (32–34), Porter believes that the four stages of regional competitive advantage are: factor-driven, investment-driven, innovation-driven, and wealth-driven. The stage of urban development will affect urban public health, so we choose *per capita* GDP (*Gdpp*) and the proportion of the secondary and tertiary industries (*Ttsr*) as control variables to characterize the stage of urban development. The second is urban form, so we choose urban construction land area (*Urss*) and urban population density (*Urbd*) as control variables. The third is human capital (*Hcap*), which measures urban human capital using the number of college students per 10,000 people. Fourth, openness to the outside world (*Open*), which is characterized by travel activity (the ratio of total urban passenger traffic to the total urban population).

### 2.5 Data sources

This paper takes 280 cities in China as the research object, and the original data for the indicators are all taken from the corresponding years’ “China Health Statistics Yearbook,” “China City Statistical Yearbook,” EPS data platform, Columbia University’s Social Economic Data and Application Center. For individual missing data, trend prediction and moving average methods are used to fill in the gaps.

## 3 Empirical results and analysis

After conducting an F-test on the “city” dummy variable, it was found that individual effects exist at a 1% significance level, rejecting the null hypothesis that there are no individual effects. Considering that models with individual effects are divided into fixed-effect and random-effect models, after conducting a Hausman test, the *p*-value is close to 0, thus accepting the null hypothesis that the fixed-effect model is selected. In summary, the regression equation constructed in this paper is a two-way fixed effects model. The two-way fixed effect model can consider both individual and time dimensions of fixed effect, which improves the explanatory power of the model. Secondly, the model can deal with heteroscedasticity and sequence correlation problems, which improves the accuracy of estimation.

### 3.1 Benchmark regression results

First, we use least squares regression to measure the correlation between economic agglomeration and urban public health before and after adding control variables. By comparing the degree of dispersion and aggregation of variables, it is found that there is a significant positive correlation between economic agglomeration and urban public health. Columns (1, 2) of Table 1 present the regression results for the baseline model’s bidirectional random effects and fixed effects, respectively; Due to the large variance of urban public health indicators, it is necessary to delete more extreme samples to ensure the robustness of the regression results. Column (3) of Table 1 shows the regression results after removing the top 10% samples of urban public health; Using the B-P test and (35) method, we tested for heteroskedasticity and cross-sectional correlation, respectively. The results showed that the equation had heteroskedasticity and cross-sectional correlation problems at a 1% significance level, which would reduce the authenticity and validity of the statistical results. Therefore, we selected the Driscoll-Kraay standard error, which addresses cross-sectional correlation and heteroskedasticity issues, instead of the standard *t*-test for significance testing. The results are shown in column (4) of Table 1.

**Table 1:** Benchmark regression results.

Variables	(1) *Re*	(2) *Fe*	(3) Exclude extreme values	(4) *D-K*	(5) *IV*	(6) *GMM*
*Urbh*	38.660*** (19.26)	33.145*** (14.56)	14.678*** (15.34)	34.456*** (3.56)	153.43*** (23.45)	98.564*** (7.32)
*Gdpp*	3.45e-04***(5.36)	3.78e-04*** (5.67)	5.35e-05*** (5.65)	3.87e-04*** (8.76)	-3.77e-05 (−0.35)	7.76e-05*** (1.66)
*Ttsr*	−0.067* (−2.34)	−0.056 (−0.77)	−0.014** (−2.23)	−0.046*** (−8.56)	−0.023 (−0.65)	−0.015 (−0.56)
*Urss*	−8.065 (−1.12)	−6.344 (−0.87)	−2.543*** (−3.12)	−6.234*** (−3.56)	−10.134*** (−3.32)	−10.112*** (−2.54)
*Urbd*	1.354 (0.84)	0.675 (0.45)	0.267 (1.49)	0.723 (0.05)	0.343 (0.58)	−0.253 (−0.36)
*Hcap*	0.146 (0.85)	0.185 (1.03)	0.134*** (6.63)	0.187*** (3.24)	0.175*** (2.45)	0.154* (1.43)
*Open*	−0.232*** (−3.23)	−0.164** (−2.16)	0.002 (0.34)	−0.145 (−0.42)	−0.092*** (−3.12)	−0.054 (−1.24)
time effect	Control	Control	Control	Control	Control	Control
individual effect	Control	Control	Control	Control	Control	Control
constant	8.345*** (2.76)	3.894 (0.87)	0.588** (1.87)	0	0	0
*R* ^2^	0.4492	0.4678	0.4671	0.4772	0.7682	0.4123

The *t*-value is in parentheses, and ***, **, and * indicate significance at the 1, 5, and 10% levels, respectively.

In addition, as the improvement of urban public health level will promote economic agglomeration, economic agglomeration may in turn improve urban public health level. This endogenous problem caused by two-way causality needs to be solved using instrumental variable two-stage least squares regression and generalized method of moments estimation methods. First, we use instrumental variables to deal with endogenous problems, and select exogenous instrumental variables to deal with potential endogenous problems. GMM does not need to know the exact distribution information of the random error term, allowing the random error term to have heteroscedasticity and sequence correlation, so the obtained parameter estimators are more efficient than other parameter estimation methods. The ratio of the length to the width of the city is *Lrb*. The closer this value is to 1, the higher the degree of equilibrium in the spatial diffusion of the city around the city center point. It also represents a more homogeneous geographical environment around the city, with the potential to develop into a high-level city. This indicator does not necessarily have a direct causal relationship with public health, so this paper uses (*Lrb*-1)^2^, a variable that measures the degree of urban equilibrium, as one of the instrumental variables, and uses *Lrb* as the other instrumental variable. These two instrumental variables passed the instrumental variable tests for the Cragg-Donald Wald F statistic, Sargan statistic, and Anderson canon. Corr. LM statistic. Column (5) of Table 2 shows the regression results of IV-2SLS. Secondly, we used the system GMM method and selected the lagged two and three periods of economic agglomeration as instrumental variables for two-stage least squares regression. The regression results are shown in column (6) of Table 2.

**Table 2:** Results of robustness test.

Variables	(1) East China	(2) Central China	(3) West china	(4) Northeast China	(5) Medical Pilot City	(6) Non-medical pilot cities	(7) Lag one period	(8) Microdata
*Urbh*	45.654*** (11.43)	14.335*** (6.45)	34.564*** (15.54)	62.654*** (11.35)	31.64*** (7.54)	22.65*** (11.43)	30.675*** (15.65)	401.34*** (5.32)
*Gdpp*	2.29e-04*** (7.56)	3.68e-04*** (7.53)	−3.86e-05 (0.156)	1.83e-05 (0.43)	1.80e-05** (2.23)	1.62e-05** (9.54)	1.97e-05*** (9.34)	1.91e-03 (−0.76)
*Tsr*	−0.062* (−1.89)	−0.062*** (−3.18)	−0.038 (−2.65)	0.043* (1.86)	0.042 (0.52)	−0.052*** (−5.42)	−0.052*** (−3.86)	−1.542 (−0.72)
*Urss*	−10.872*** (−3.87)	−4.892 (−1.42)	−4.652*** (−1.36)	−2.672 (−0.38)	−14.89** (−2.16)	−3.173* (−1.78)	−6.472*** (−3.72)	−1.787** (−2.32)
*Urbd*	−0.887 (−1.43)	−0.198 (−0.38)	0.078 (0.15)	0.328 (0.56)	−2.127 (−1.53)	0.189 (−0.56)	0.016 (0.04)	−1.365 (−1.23)
*Hcap*	0.329*** (3.87)	0.187*** (3.53)	−0.087 (−1.53)	−0.032 (0.28)	0.128 (0.58)	0.112*** (3.43)	0.065* (1.54)	−4.234 (−0.26)
*Open*	0.058 (1.87)	0.051* (1.76)	−0.042** (−2.33)	−0.032 (−0.95)	0.018 (0.17)	−0.008 (−0.69)	0.011 (0.66)	−2.65e-04** (−2.45)
time effect	Control	Control	Control	Control	Control	Control	Control	Control
individual effect	Control	Control	Control	Control	Control	Control	Control	Control
constant	6.165** (2.43)	4.135** (2.16)	4.765*** (3.34)	−3.786* (−1.69)	−1.652 (−0.18)	4.886*** (5.36)	4.454*** (3.74)	132.8 (0.58)
*R* ^2^	0.5943	0.4015	0.5505	0.6006	0.6488	0.4082	0.4360	0.1228

The *t*-value is in parentheses, and ***, **, and * indicate significance at the 1, 5, and 10% levels, respectively.

In the models (1–6) in Table 1, economic agglomeration has a significant promoting effect on public health levels. In models (1, 2, and 4), a 1% increase in economic agglomeration variables can improve urban public health by approximately 0.34 units. In models (3, 5, and 6), the positive and negative signs and significance of key explanatory variables in the regression results without extreme values and using instrumental variables remain unchanged, indicating that under different conditions, the role of economic agglomeration in improving urban public health is not covered by other influencing factors, that is, economic agglomeration is an important factor in improving urban public health. Economic factors are characterized by scarcity and profit-seeking, and will flow from areas with low marginal returns to areas with high marginal returns. This “selecting the best” mechanism will encourage economic factors to flow to higher-tier cities in order to increase their own value and marginal output efficiency, optimize the efficiency of urban public resource allocation, and thus have a positive impact on urban public health.

In terms of control variables, *per capita* gross domestic product (*Gdpp*) and human capital (*Hcap*) both significantly promote urban public health. This is mainly due to the fact that improving economic development and increasing human capital will inevitably attract more public health factors to gather, which is beneficial to the improvement of public health. The urban construction land area (*Urss*) and the proportion of the secondary and tertiary industries (*Ttsr*) have a suppressing effect on urban public health, indicating that the disorderly expansion of urban construction land area will reduce the efficiency of land resource allocation (26), while foreign investment will to some extent inhibit the improvement of urban public health. There is a non-significant positive correlation between urban population density (*Urbd*) and urban public health, indicating that most cities in China still need to increase the introduction of medical technicians to promote the improvement of public health through the increase of urban “doctor density.”The impact of opening up on urban public health is not robust, possibly because the level of urban public health is mainly supported by local governments, and the correlation with opening up is not strong.

### 3.2 Robust test

This paper will test the robustness of the regression results from four perspectives: regional heterogeneity, policy impact of pilot cities for compact urban medical groups, lag effect, and micro-sample.

First, consider the impact of regional heterogeneity on the results. The robustness test was conducted using a sub-regional regression approach. Columns (1–4) in Table 2 present the regression results for the four major regions of eastern, central, western, and northeastern China. Through comparative analysis, it can be seen that although the impact coefficients of economic agglomeration on public health in the regression results of the four major sectors are all positive and the significance level is 1%, there is heterogeneity in the promotion effect of economic agglomeration on public health among the four major sectors.

Second, control the policy impact of pilot cities for closely controlled urban medical groups. Since 2013, China has gradually established pilot cities for the construction of compact urban medical groups. These groups are internally composed of leading hospitals and member units. In principle, the leading hospitals are municipal-level, district-level, and tertiary comprehensive hospitals (including traditional Chinese medicine hospitals). At least one-third of the outpatient number sources and one-fourth of the inpatient beds should be provided to family doctor contract service teams or grassroots medical and health institutions. Contracted residents who are referred by grassroots can have priority in treatment, examination, and hospitalization. The leading hospital focuses on providing diagnosis and treatment services for acute and critical illnesses and difficult and complex diseases, and is responsible for receiving patients transferred from other hospitals and referring patients who meet the criteria for transfer to member units in an orderly manner. In principle, member units should include at least a second-tier general hospital or a medical institution capable of providing common and chronic disease diagnosis and treatment, emergency and critical care rescue, and continuity of care for patients referred by leading hospitals. At the same time, the close-knit urban medical group will coordinate the construction of resource sharing centers for medical testing, medical imaging, electrocardiographic diagnosis, pathology, disinfection, and supply, etc., to achieve mutual recognition of inspection and testing results within the close-knit urban medical group, establish a telemedicine collaboration network covering all units of the medical consortium, and improve the efficiency of medical resource allocation and use. It is worth noting that most of the samples selected for the pilot cities of the compact urban medical group are high-level cities. Therefore, the non-randomness of the sample selection in this policy will lead to the improvement of urban public health not from the improvement of economic agglomeration, but from the consequences of the policy impact, resulting in a decrease in the credibility of the results. Therefore, in order to control the impact of the policy of pilot cities for compact urban medical groups, we will conduct a regression analysis between the pilot cities for compact urban medical groups and the pilot cities for non-compact urban medical groups. As shown in columns (5, 6) of Table 2, the promotion of economic agglomeration in both pilot cities of compact urban medical groups and pilot cities of non-compact urban medical groups has a significant positive impact on urban public health.

Third, consider the lag effect. The improvement of economic agglomeration may take some time before it has an impact on urban public health, and economic agglomeration reflects the long-term changes in urban development, while the level of urban public health reflects short-term fluctuations. There may be an effect estimation bias in the direct regression between the two. Therefore, in order to avoid this situation, all explanatory variables are regressed with a lag of one period. As shown in column (7) of Table 2, there is no fundamental change in the positive role of economic agglomeration in promoting urban public health.

Fourth, we use micro-sample data to verify. Match the database of public hospitals with urban panel data, and select the number of urban public hospitals as the dependent variable to conduct a regression analysis. The results are shown in column (8) of Table 2, which is consistent with the results of the benchmark regression. The increase in economic agglomeration will promote the increase in the number of urban public hospitals. In addition, the coefficients and significance of the control variables are basically consistent with the regression results in Table 1. It can be seen that the sign and significance of the key explanatory variables have not changed under different empirical model analyses using instrumental variables, indicating that under different conditions, the role of economic agglomeration in urban public health has not been covered by other influencing factors, that is, economic agglomeration is an important factor in improving public health.

### 3.3 Granger causality identification

The Granger causality test is one of the classic methods for testing causal relationships in time series models. Based on this, Dumiterescu and Hurlin (36) proposed an idea that is considered to extend the Granger causality test to panel models, which has been widely applied in empirical testing (36–39). This paper aims to study the causal relationship between economic agglomeration and urban public health, and the regression equation established is as follows:


(2) $$\mathrm{Phealt}h_{\mathrm{it}}=\overset{N}{\underset{i=1}{\sum }}\lambda_{j}L_{j}.Urbh_{\mathrm{it}}+\overset{N}{\underset{j=1}{\sum }}\lambda_{j}L_{j}.\mathrm{Phealt}h_{\mathrm{it}}+\lambda_{0}+\zeta_{\mathrm{it}}$$

Where *j* is the optimal lag order, and according to the principle of minimizing the AIC and BIC information criteria, two periods are selected as the lag order. The regression results are shown in Table 3. Columns (1, 2) are the ordinary panel VAR models, and columns (3, 4) are the regression results after taking the difference between the independent variable and the dependent variable, aiming to reduce the possible impact of unit roots on the regression results. Columns (4, 5) are the results after controlling for the fixed effect of time, aiming to reduce the impact of time trends on the regression results. It can be found that although the urban public health and economic agglomeration in columns (1, 2) are Granger causes of each other at the 1% significance level. However, in columns (3–6) where the basic assumptions of no unit root and no time trend affecting the results are relaxed, economic agglomeration is a Granger cause of urban public health at the 1% significance level, but urban public health is not a Granger cause of economic agglomeration. Therefore, the improvement of economic agglomeration is a prerequisite for the improvement of public health, but the opposite is not true. It can be seen that although the improvement of urban public health will promote urban economic growth, it will also promote economic agglomeration. However, the improvement of urban public health level is not spontaneous, it requires the investment of capital and high-skilled medical workers, and both of them are improved with the increase of economic agglomeration. Therefore, the premise of improving urban public health is the accumulation of public health factors and the agglomeration of medical resources brought about by economic agglomeration.

**Table 3:** Granger causality results.

Variables	(1) *Inoe*	(2) *Urbh*	(3) *Inoe*	(4) *Urbh*	(5) *Inoe*	(6) *Urbh*
*L.Inoe*	1.192*** (41.66)	−0.002*** (−2.78)	0.287*** (11.98)	0.001 (1.06)	1.412*** (42.66)	−0.001 (−1.87)
*L2.Inoe*	−0.048 (−1.42)	0.007*** (9.87)	0.033 (0.62)	0.007 (1.32)	−0.098*** (−2.78)	0.004 (1.32)
*L.Urbh*	4.983*** (5.82)	0.453*** (21.54)	7.387*** (5.65)	−0.653*** (−22.46)	2.321*** (2.52)	0.531*** (23.43)
*L2.Urbh*	−2.872*** (−2.82)	0.378*** (16.87)	3.324** (2.32)	−0.282*** (−9.65)	−0.667 (−0.56)	0.427*** (14.87)
Time Trend	–	–	–	–	Control	Control
constant	0.054*** (3.78)	0.001*** (6.87)	0.119*** (9.18)	0.003*** (8.87)	0.398*** (12.53)	−0.001 (0.44)
*R^2^*	0.8762	0.7253	0.1097	0.2098	0.9092	0.7562
Joint significance *P* value	Remarkable	Significance	Significance	Not significance	Significance	Not significance

The *t*-value is in parentheses, and ***, **, and * indicate significance at the 1, 5, and 10% levels, respectively.

## 4 Conclusions and policy recommendations

1. The improvement of China’s economic agglomeration can significantly promote urban public health. The Granger causality test results show that economic agglomeration is a prerequisite for urban public health, but the converse is not true. This means that the prerequisite for a city to become a regional public health center is to first become a regional central city. Only large cities can better promote public health, and even increasing medical investment in small and medium-sized cities cannot effectively promote their economic agglomeration.
2. There is regional heterogeneity in the promotion effect of economic agglomeration on urban public health. The promotion effect of economic agglomeration in the northeast region on urban public health is the largest compared to other regions, followed by the eastern region, the western region, and the central region. What is more worth emphasizing is the population growth in the coastal cities of eastern China, as well as the population growth in the suburbs and new towns of cities, with a large number of immigrants as the main population. Due to the constraints of the household registration system and the associated social welfare system, these migrants do not have the same equal access to health and health services as local citizens. The vast majority of them do not enjoy medical insurance that urban residents have, and it is difficult for them to obtain high-quality health and health services that urban residents can obtain. The differential rights of migrant population in health and health services is a microcosm of their status in cities, and also undermines the public nature of urban health and public services. The promotion of pilot cities of close urban medical groups and non-close urban medical groups has a significant promoting effect on urban public health. The promotion of economic agglomeration in pilot cities of close urban medical groups has a greater promoting effect on public health than that in pilot cities of non-close urban medical groups. At the same time, empirical results based on micro-sample data show that the increase in economic agglomeration also promotes the increase in the number of urban public hospitals.
3. As an important part of patriotic health work in the new era, the construction of healthy cities is an important means to further implement the Healthy China strategy and promote the construction of a healthy China. A healthy city refers to an organic whole composed of healthy people, healthy environment and healthy society, with people’s health as the center and the protection of the health of the general public as the focus, from urban planning, construction to management. In the future, we should take the construction of healthy cities as an important starting point for promoting the construction of a healthy China, ensure the demand for land for public facilities related to health, improve the relevant public facility systems, layouts, and standards, integrate health into the whole process of urban and rural planning, construction, and governance, and promote the coordinated development of cities and people’s health. Efforts to provide universal health public services in the process of urbanization should become an important policy orientation to reduce urban internal differentiation and improve the quality of urban life.

## Data Availability

Publicly available datasets were analyzed in this study. This data can be found at: http://www.tjcn.org.
